# Kinetic Pathway of Pyrophosphorolysis by a Retrotransposon Reverse Transcriptase

**DOI:** 10.1371/journal.pone.0001389

**Published:** 2008-01-02

**Authors:** Manjula Pandey, Smita S. Patel, Abram Gabriel

**Affiliations:** 1 Department of Molecular Biology and Biochemistry, Rutgers University, Piscataway, New Jersey, United States of America; 2 Department of Biochemistry, University of Medicine and Dentistry of New Jersey, Piscataway, New Jersey, United States of America; University of Massachusetts, United States of America

## Abstract

DNA and RNA polymerases use a common phosphoryl transfer mechanism for base addition that requires two or three acidic amino acid residues at their active sites. We previously showed, for the reverse transcriptase (RT) encoded by the yeast retrotransposon Ty1, that one of the three conserved active site aspartates (D_211_) can be substituted by asparagine and still retain *in vitro* polymerase activity, although *in vivo* transposition is lost. Transposition is partially restored by second site suppressor mutations in the RNAse H domain. The novel properties of this amino acid substitution led us to express the WT and D_211_N mutant enzymes, and study their pre-steady state kinetic parameters. We found that the *k_pol_* was reduced by a factor of 223 in the mutant, although the *K_d_* for nucleotide binding was unaltered. Further, the mutant enzyme had a marked preference for Mn^2+^ over Mg^2+^. To better understand the functions of this residue within the Ty1 RT active site, we have now examined the *in vitro* properties of WT and D_211_N mutant Ty1 RTs in carrying out pyrophosphorolysis, the reverse reaction to polymerization, where pyrophosphate is the substrate and dNTPs are the product. We find that pyrophosphorolysis is efficient only when the base-paired primer template region is >14 bases, and that activity increases when the primer end is blunt-ended or recessed by only a few bases. Using pre-steady state kinetic analysis, we find that the rate of pyrophosphorolysis (*k_pyro_*) in the D_211_N mutant is nearly 320 fold lower than the WT enzyme, and that the mutant enzyme has an ∼170 fold lower apparent *K_d_* for pyrophosphate. These findings indicate that subtle substrate differences can strongly affect the enzyme's ability to properly position the primer-end to carry out pyrophosphorolysis. Further the kinetic data suggests that the D_211_ residue has a role in pyrophosphate binding and release, which could affect polymerase translocation, and help explain the D_211_N mutant's transposition defect.

## Introduction

Template dependent nucleotide polymerases use a remarkably well conserved two metal ion dependent phosphoryl transferase mechanism for nucleotide addition. Templates, primer-ends and nucleotides are positioned at the active site, the 3′ hydroxyl of the primer-end attacks the α-phosphate of the incoming dNTP, pyrophosphate (PPi) is released and the primer-end translocates to allow for the addition of the next nucleotide [1]. A conserved structural feature of polymerase active sites is the presence of two or three acidic residues (depending on the class of polymerases) which coordinate the positioning of the metal ions [2].

Reverse transcriptases (RTs) are a class of RNA or DNA- dependentent DNA polymerases first discovered within retroviral particles [3], [4]. They are essential for retroviral replication, and copy the genomic RNA into double stranded DNA that can then integrate into a new genomic location[5]. RTs, or the genes encoding them, have subsequently been discovered in a wide range of endogenous retrotransposons and in some infectious DNA viruses, where they are used for element replication, as well as in telomerases, that use a short RNA template to create terminal DNA repeats at chromosome ends. All RTs contain three conservatively spaced aspartates, two of which form the canonical “YXDD” sequence [6], [7]. Early studies with retroviral RTs showed that mutation of any of these aspartates severely disrupts polymerase function *in vitro*, and eliminates the biological activity of the affected enzyme [8], [9]. In previous studies of the RT encoded by the yeast retrotransposon Ty1, we found that a substitution of the second D in the YXDD box (D_211_N), had surprisingly little effect on the *in vitro* ability of the enzyme to add nucleotides to homopolymer substrates, although a D_211_E substitution as well as substitution of either of the other two aspartates (D_129_ and D_210_) completely blocked polymerization [10].

While the D_211_N mutation completely inhibits transposition, we also identified second site suppressor mutations in the RNAse H domain of Ty1 RT, which restored 5–10% of the D_211_N mutant's transposition capability [10]. Although this was the first reported case of a retrotransposon with apparently loosened requirements for the acidic residues in the active site, there is now a report that a different, unrelated retrotransposon RT from yeast (Ty3) has similar if not even more extreme flexibility at these sites [11].

Because of the unusual properties of the mutant Ty1 RT, we undertook a pre-steady state analysis of single base incorporation to compare the kinetic parameters of the two enzymes, determine the nature of the defect in the D_211_N mutant, and to better understand the role of this residue in polymerization and transposition. Our results indicated that the rate constant for single nucleotide addition (*k_pol_*) was reduced 223 fold for the mutant enzyme relative to WT, but that nucleotide binding was unaffected. Each enzyme showed distinctive patterns of pausing during polymerization, suggesting differences in translocation or PPi removal for the two enzymes. Further, the two enzymes showed marked differences in utilization of Mg^2+^ versus Mn^2+^ , suggesting a role for the D_211_ side chain in binding the metal involved in nucleophilic attack [12].

Pyrophosphorolysis is the reverse of the polymerization reaction, in which the terminal base at a primer-end is excised in the presence of a properly positioned PPi group, generating a dNTP and a primer one base shorter than its initial length. Pyrophosphorolysis has been useful in studying the events occurring at the polymerase active site. In particular it has become apparent that excision of incorporated nucleotide analogue chain-terminators is an important drug resistance mechanism utilized *in vivo* by mutant versions of HIV1 RT, and possibly other viral polymerases [13]–[15]. This process is mechanistically analogous to pyrophosphorolysis, except that ATP is the PPi donor [16], [17]. Analysis of this phenomenon, and the effects of various analogs and mutations in HIV1 RT on the relative efficiency of this process, has helped to explicate the events at the active site that result in pyrophosphorolysis and the related phenomenon of translocation and processive synthesis [18], [19]. The primer-end in the active site of a polymerase can be present in one of two positions, referred to as the P and N sites. With the primer-end in the P site, an incoming nucleotide can bind to the N site, and chemistry can occur between the 3′ hydroxyl and the alpha phosphate of the dNTP. Post-chemistry, the primer-end resides in the N site, which also houses the PPi group. The chemical step at the active site corresponds to the closing down of a finger domain over the active site, leading to a “closed” conformation [20]. Translocation then involves the movement of the primer-end from the N site back to the P site, accompanied by release of the PPi from the N site pocket. This corresponds to a switch back to an “open” conformation. An important insight from the work on HIV-1 nucleotide analog resistance mutants is that the equilibrium between the primer-end at the P site versus the N site is an important determinant in the ability to carry out excision, since this reaction can only occur if the primer-end is positioned at the N site, which may not be a favorable placement.

Given the unusual observations regarding the biochemical properties of the D_211_N mutant in polymerization, and the importance of primer-end placement in the active site, we set out to determine how the substitution would affect the process of pyrophosphorolysis. Previous studies on HIV1 RT and Ty3 RT have shown that certain mutations do not necessarily affect polymerization and pyrophosphorolysis equally, suggesting that binding or positioning of the substrates in one direction or the other can be differentially affected [11], [21]. Since this could give further insights into the functions of the D_211_ residue, we carried out a series of experiments to characterize determinants of pyrophosphorolysis for the WT and mutant enzyme, using either Mg^2+^ or Mn^2+ ^as the divalent cation. We then measured the apparent *K_d_* for PPi and the *k_pyro_* using pre-steady state kinetics. This study of pyrophosphorolysis, along with our previous examination of polymerization, form the frame work for future understanding of the mechanistic basis of the activity of the Ty1 RT second site suppressor mutants.

## Results

### D_211 _N mutant RT enzyme is defective in pyrophosphorolysis

**Figure 1:**
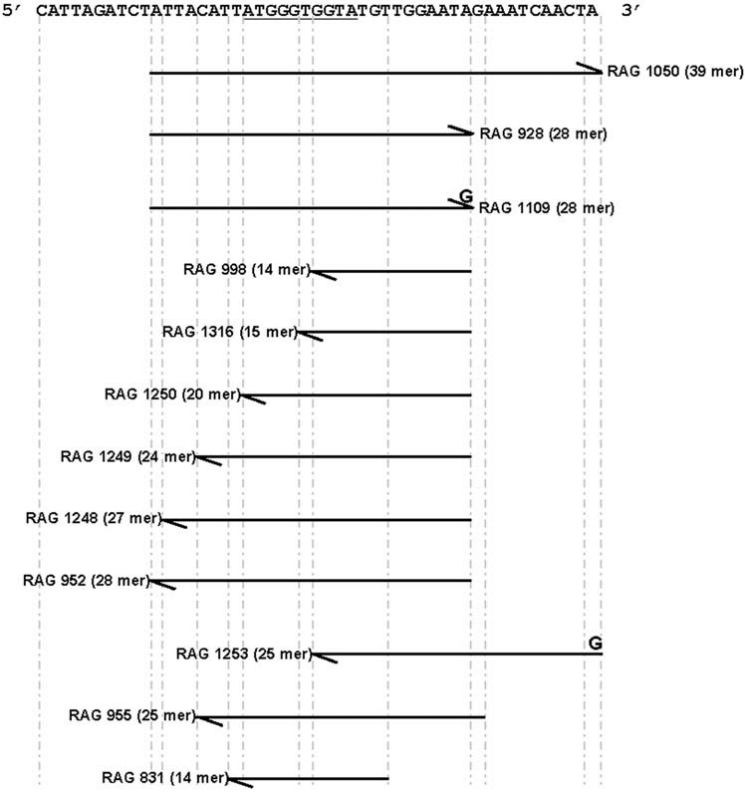
DNA sequences used in this study. Sequence shows the PPT region of Ty1. All the oligonucleotides (RAGs) used in this study correspond either to the plus or minus strand of this sequence, depending on the direction of the arrow. The relative positions of the oligonucleotides and modifications are shown in this figure.

**Figure 2:**
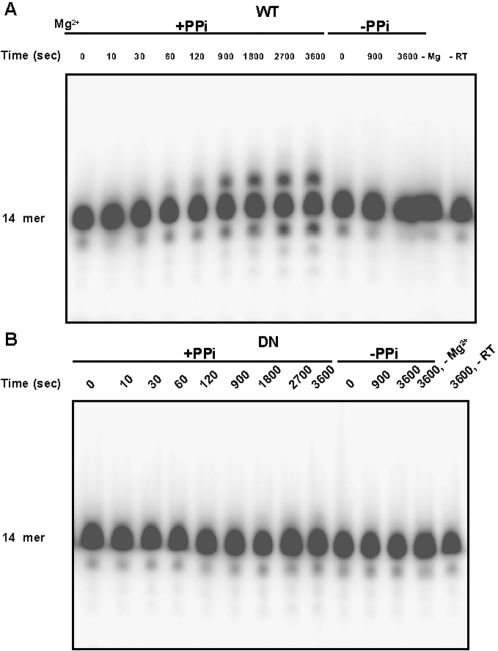
Pyrophosphorolysis by WT and D_211_N Ty1 RT of a recessed primer terminus. Pyrophosphorolysis reactions by WT or mutant D_211_N Ty1 RT in the presence of 10 mM Mg^2+ ^and 1 mM sodium pyrophosphate were analyzed by *in vitro* assay as described under materials and methods using 5′ ^32^P-end labeled 14-mer/28-mer substrate (RAG*998/RAG 928, Fig. 1. Labeled end is depicted by *). A, represents time course of WT reactions in seconds with or without PPi. B represents time course of D_211_N reactions in seconds with or without PPi. Control reactions in both panels are in the absence of Mg^2+ ^or enzyme.

We initially compared the time course of pyrophosphorolysis (0–3600 sec) for the two enzymes using our previous ^32^P-labeled 14-mer/28-mer DNA/DNA substrate [12] (RAG*998/928, Fig. 1) in the presence of 1 mM sodium pyrophosphate and 10 mM Mg^2+^. The behavior of the two enzymes was quite distinct under substrate excess conditions (Fig. 2). The WT enzyme was able to excise a base from ∼10% of the labeled primer within 60 seconds. The fraction of excised substrate increased to ∼17% over the time course (Fig. 2A). Time points beyond 120 seconds also showed base addition. Under the same conditions, pyrophosphorolysis with the D_211_N RT was insignificant, amounting to <1% of the total substrate at the longest time point (Fig. 2B). We did not observe any base addition with the mutant enzyme either. Since there are no nucleoside triphosphates initially present in the in vitro reaction, the presence of base addition in WT RT reactions (Fig. 2A) suggested that nucleoside triphosphates were being generated by pyrophosphorolysis of the unlabeled strand of the double stranded substrate, and subsequently used for polymerization. This is consistent with the observation that the excised base from the unlabeled strand (dATP) is complementary to the next base beyond the primer, for this primer/template combination.

### Pyrophosphorolysis on the 5′ blunt end

**Figure 3:**
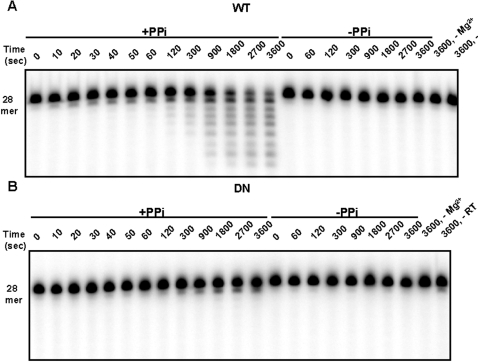
Pyrophosphorolysis by WT and D_211_N Ty1 RT of a blunt-ended primer terminus. Pyrophosphorolysis reactions of WT or mutant D_211_N Ty1 RT were carried out under same conditions as Fig. 2 in the presence of 10 mM Mg^2+^, using 5′ ^32^P-end labeled 28-mer/14-mer substrate (RAG*928/998, Fig. 1). A, represents time course of WT reactions in seconds in the presence or absence of PPi. B, represents time course of D_211_N reactions with or without PPi. Control reactions are in the absence of Mg^2+ ^or enzyme.

To determine whether pyrophosphorolysis is simultaneously occurring at the other end of the 14-mer/28-mer DNA/DNA substrate, we labeled the 5′ end of the 28-mer oligonucleotide with ^32^P, instead of the 14-mer, and repeated the time course reactions with this new substrate (RAG 998/*928, Fig. 1) under the same conditions. Unexpectedly, WT reactions were much more extensive on this end of the substrate, with ∼4.2% of the total substrate excised by 10 sec. By 900 seconds almost 50% of the substrate had at least one base excised. By the end of the time course, a substantial portion of the substrate had been reduced by 8 bases (Fig. 3A). The large amount of dNTPs generated by pyrophosphorolysis of the blunt end could support the forward reaction observed in Fig. 2A. The D_211_N mutant also shows improved pyrophosphorolysis (∼14% of the total substrate by 3600 seconds) but was not nearly as robust as the WT enzyme (Fig. 3B).

The apparent preferential pyrophosphorolysis at the blunt end, and the generation of polymerization substrates made examination of the excision reaction at the recessed end too complex. To simplify, we modified the original substrate by introducing a mismatched guanosine at the 3′ end of the 28-mer template (resulting in RAG*998/1109, Fig. 1). In the presence of PPi, the WT enzyme was able to excise a base from the labeled primer without showing any forward reaction products (Fig. S1A). Therefore, a mismatch at the blunt end blocks base addition at the recessed end, presumably by inhibiting pyrophosphorolysis at the mismatched blunt end. The D_211_N mutant reactions were very poor and no pyrophosphorolysis activity was observed even at 3600 seconds (Fig. S1B).

### Primer length effects

**Figure 4:**
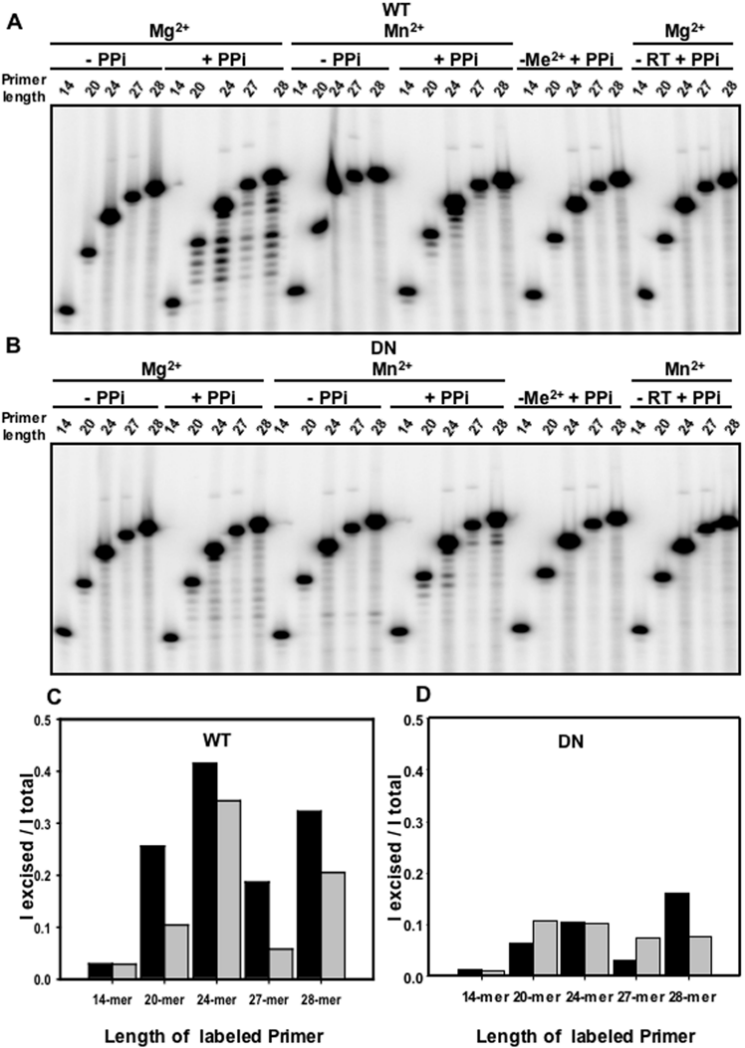
Effect of primer length on pyrophosphorolysis. Pyrophosphorolysis by WT or mutant D_211_N Ty1 RT were carried out for 60 minutes in the presence of either 10 mM Mg^2+^ or 2 mM Mn^2+^. Substrates are 5′ ^32^P-end labeled primers of lengths 14, 20, 24, 27, 28-mer (RAG 998, RAG 1250, RAG 1249, RAG 1248, RAG 952 respectively, Fig. 1), each paired with the 28′-mer template oligo (RAG 1109, Fig. 1). A, shows WT reactions with different primer length substrates in the presence or absence of PPi. B, represents D_211_N reactions with or without PPi. Control reactions on both the panels are in the absence of enzyme or Metal ^2+^. C and D represent bar graphs for WT and DN Ty1 RT reactions depicting ratios of intensities of cleaved products to the total products in reactions (I cleaved products/I total bands). Black bars are for reactions in Mg^2+^ while grey bars are for reactions in Mn^2+^.

The preceding analysis indicated that the recessed primer end of the 14-mer/28-mer or 28′-mer substrates (RAG*998/928, RAG*998/1109, Fig. 1, where “ **’** ” refers to the terminal mismatch) is a poor substrate for pyrophosphorolysis. To determine the effects of altering substrate lengths and/or the degree of primer end recessing, we used a variety of substrates (Fig. 4) that differed only by the length of the 5′-end ^32^P-labeled primer, as a 14-mer, 20-mer, 24-mer, 27-mer or 28-mer (RAGs 998, 1250,1249, 1248 and 952 respectively as in Fig. 1). Primers were annealed to the same terminally mismatched 28′-mer template. Further, we carried out the 60-minute reactions using either Mg^2+^ or Mn^2+^ as the divalent metal. For both WT and D_211_N mutant enzymes (Fig. 4A & B) pyrophosphorolysis activity significantly improved with the increase in length beyond the 14-mer in the presence of Mg^2+^. In particular, the WT enzyme was able to excise >30% of the 24-mer and 28-mer substrates (Fig. 4C). The D_211_N mutant enzyme pyrophosphorolysis activity also improved proportionately with longer primers compared to the 14-mer primer but was still defective in comparison to WT (Fig. 4C and 4D).

As previously reported [12], [22], for polymerization, the WT enzyme has a marked Mg^2+^ preference, and is inhibited by Mn^2+^, whereas the mutant enzyme prefers Mn^2+^ to Mg^2+.^ In the presence of Mg^2+^, the WT enzyme excised a larger percentage of substrates than in Mn^2+^(Fig. 4C). The D_211_N mutant enzyme showed no clear preference under these conditions (Fig. 4D). For both enzymes, the patterns of excision differ in the presence of the different metals, with a greater number of bases removed from any given excised substrate in the presence of Mg^2+^(Fig. 4A and B).

These results demonstrate that substrates with double stranded primer/template regions greater than 14 bps are better substrates for the Ty1 RT pyrophosphorolysis reaction. Of note, during processive excision, as seen in Fig. 4A, most excised products remained longer than 14-bases long, and a 14 base primer appears to be a limit to excision. Increased primer length is not the only factor associated with increased pyrophosphorolysis activity, however, since the 24-mer was the most active, a blunt 28-mer was next most active and a 20-mer was more active than the 27-mer. The Mn^2+^ results for WT mirrored those with Mg^2+^, suggesting that metal ion was not the determining factor in relative activity on different substrates.

We further explored the influence of different substrate arrangements on pyrophosphorolysis (Fig. S2). These comparisons demonstrated that the poor reaction of the original 14 mer/28 mer is not due to the length of the recessed end, since a substrate with the same recessed 14 bases, but a double stranded region extended by an additional 11 bases underwent robust pyrophosphorolysis.

### Pyrophosphorolysis processivity

**Figure 5:**
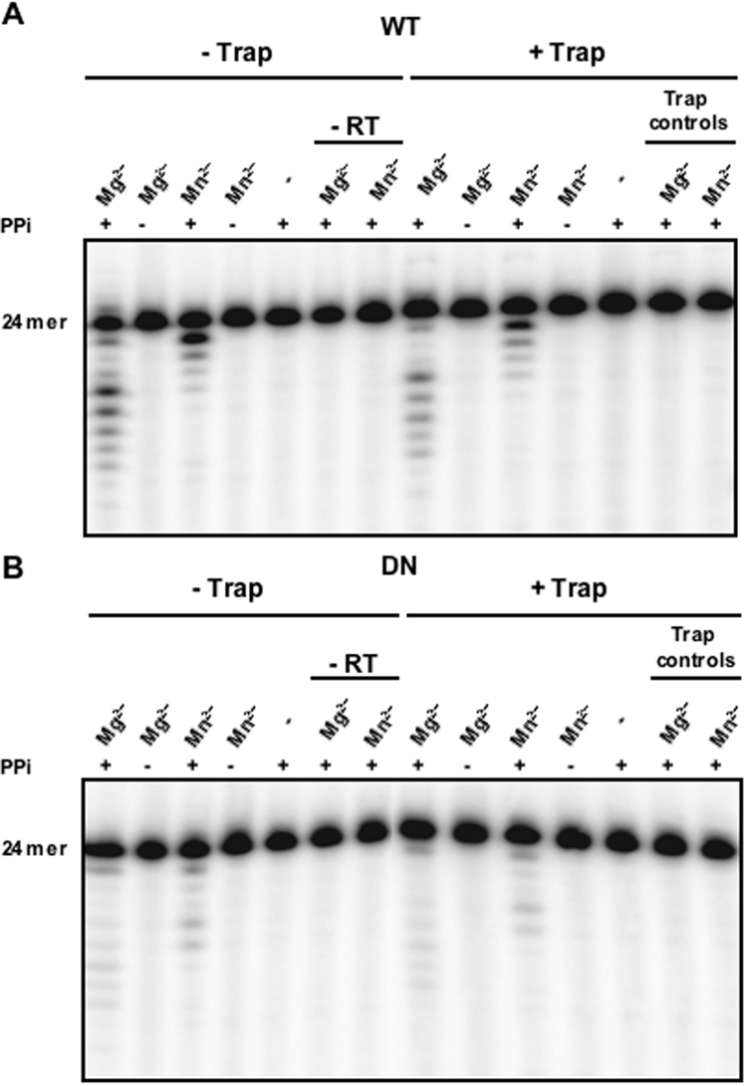
Processivity of the Ty1 pyrophosphorolysis. Pyrophosphorolysis by WT or mutant D_211_N Ty1 RT were carried out for 60 minutes in the presence or the absence of trap and in the presence of either 10 mM Mg^2+^ or 2 mM Mn^2+^. Substrates are 5′ ^32^P-end labeled substrate (RAG*1249/1109, Fig. 1). A, shows WT reactions in the presence or absence of 200 fold excess of cold 24-mer/28′-mer DNA/DNA substrate trap and with or without PPi (depicted as + and – respectively). B, similarly represents D_211_N reactions. Control reactions on both the panels are in the absence of enzymes. Trap effectiveness controls are also shown.

In our previous study [12] we found that both the WT and D_211_N mutant were highly processive for the polymerase reaction. Since we observed multiple excision products, we asked whether excision of multiple bases was processive or distributive. The reactions were carried out in the presence or absence of a trap in excess to the labeled substrate, and in the presence of Mg^2+^ or Mn^2+^. Trap effectiveness controls and control reactions without enzyme or PPi or divalent ions were also conducted. Fig. 5A shows that either in the presence or absence of trap, and in the presence of metal, the WT enzyme can excise multiple bases. This indicates that pyrophosphorolysis is processive in the presence of either divalent metal ion. The D_211_N enzyme was similarly insensitive to the presence of the trap (Fig. 5B) indicating that the D_211_N RT is also processive for pyrophosphorolysis in the presence of either divalent metal ion.

### Pre-steady-state kinetics of pyrophosphorolysis for WT and mutant D_211 _N Ty1 RT

Our steady state studies showed that the D_211_N mutant enzyme was much less proficient at pyrophosphorolysis, but did not provide a quantitative analysis of the kinetic differences. To examine this, we turned to pre-steady state single base excision kinetic analysis. Earlier, we had shown that single base addition onto the 14-mer/28-mer RAG 998/928 substrate was ∼223 fold slower for the mutant enzyme than the WT RT, but that dNTP binding was only minimally affected [12]. To measure the reverse reaction of polymerization, we generated the corresponding 15-mer/28′-mer reverse substrate, RAG*1316/1109 (Fig. 1), which was comparable to the original 14-mer/28-mer but had the next base (A) added to the primer-end. The only difference between our previous polymerization substrate and our current pyrophosphorolysis substrate was a single base mispair at the opposite end to eliminate pyrophosphorolysis of the blunt end substrate. As this change is not involved in the measured reaction, and is distant from the substrate at the active site, it should not affect the derived kinetic parameters for either polymerization or pyrophosphorolysis.

We first compared pyrophosphorolysis with the RAG*998/1109 (14-mer/28′-mer) and RAG*1316/1109 (15-mer/28′-mer) substrates using WT Ty1 RT, under the enzyme excess conditions to be used for the pre-steady state studies. Whereas, only 6.8% of the 14-mer primer was excised by at least one base, 50.1% of the 15-mer primer was excised in the presence of 1 mM PPi during a 30 minute reaction (data not shown). Therefore, this 15-mer/28′-mer substrate could be used to compare with our previous single correct base incorporation kinetics. Here, we are interested in determining the parameters of Ty1 RT pyrophosphorolysis, *k_pyro_* and *K_d_* for PPi.

**Figure 6:**
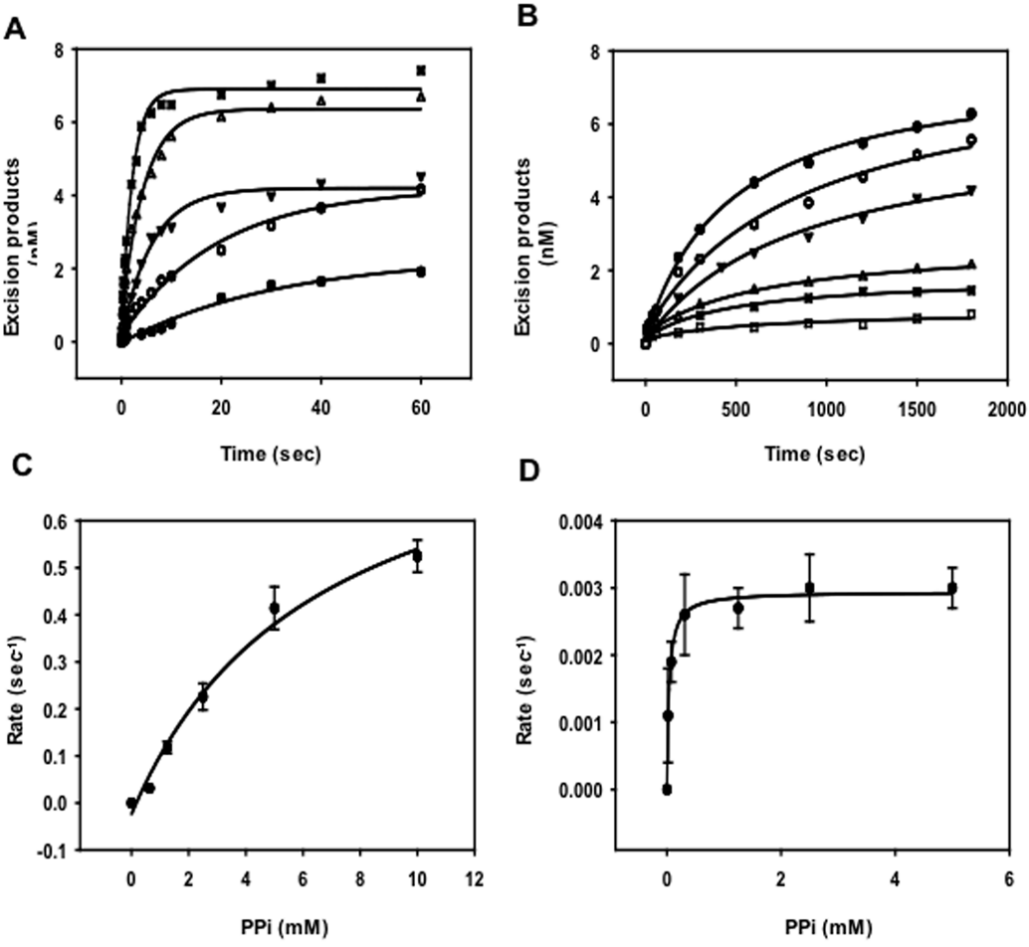
Concentration dependence of the rate of pyrophosphorolysis by WT and D_211_N Ty1 RT. A and B show time courses of a single turnover at various sodium pyrophosphate concentrations: 0.6 mM (closed circle), 1.25 mM (open circle), 2.5 mM (closed inverted triangle), 5 mM (open triangle) and 10 mM (closed square) for WT (A) and 0.02 mM (open square), 0.08 mM (closed square), 0.3 mM (open triangle), 1.25 mM (closed inverted triangle), 2.5 mM (open circle) and 5 mM (closed circle) for the D_211_N enzyme (B) respectively. A 5′ ^32^P-end labeled substrate 15-mer/28′-mer (RAG*1316/1109, Fig. 1) was used for the experiment. For WT, samples were taken at 0, 0.1, 0.2, 0.4, 0.6, 0.8, 1, 1.5, 2, 3, 4, 6, 8, 10, 20, 30, 60 second time points. For the mutant, samples were taken at 5 or 10 second intervals for the first minute and then every minute for the next 4 minutes. C and D show pre-steady-state rate dependence of nucleotide cleavage on PPi concentration for WT and mutant respectively, based on the rates derived from Equation 1 (see Experimental Procedures) in A and B. Curves represent the best fits to Equation 2 (see Experimental Procedures).

The 5′ end-labeled 15-mer primer was annealed to a 28′-mer template, mixed with RT and varying concentrations of sodium pyrophosphate and reactions were initiated by the addition of Mg^2+^. Reactions were quenched at various times and products were visualized and quantitated. The exponential rate constants were derived by fitting the data to equation 1, as shown by the solid lines in Fig. 6 A & B. Note that the concentration of excision products (i.e. the amplitude) in both Fig. 6A and 6B increases as a function of the initial PPi concentration, indicating that the reaction has come to equilibrium at the active site. This phenomenon represents the balance between the thermodynamically unfavorable pyrophosphorolyis reaction and the much more favorable polymerization reaction that uses the dNTP generated at the active site. As the concentration of PPi increases, the equilibrium is shifted towards pyrophosphorolysis.

The derived exponential rate constants were plotted against their respective PPi concentrations and the curve was fitted to a hyperbolic equation (Equation 2 and Fig. 6C & D *)* from which *K_d_* and *k_pyro_* values were derived. In deriving *K_d_*, we assumed that PPi is in rapid equilibrium with 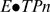. In this way, we found apparent *K_d_* values of 6.52±2.43 mM and 0.039±0.007 mM and *k_pyro_* values 0.93±0.15 sec^−1^ and 0.0029±0.0001 sec^−1^ respectively for the WT and mutant D_211_N enzymes. These correspond to an ∼170 fold greater binding affinity of PPi for the mutant enzyme, and an ∼320 fold decrease in the rate of pyrophosphorolysis for this enzyme. The derived catalytic efficiency was only ∼2 fold lower for the mutant RT enzyme compared to the WT enzyme. In our previous study, using similar approaches for the forward polymerization reaction, we found apparent *K_d_* values for dATP of 2.4±0.4microM and 1.0±0.04 microM and *k_pol_* values of 7.8±0.4 sec^−1^ and 0.035±0.002 sec^−1^ respectively for the WT and mutant D_211_N enzymes. These values indicate a similar binding affinity of dATP for the two enzymes, but an ∼100 fold lower catalytic efficiency for the mutant enzyme. Thus while both the forward and reverse reactions are significantly slower for the mutant enzyme than the WT enzyme, the differences in binding affinity and catalytic efficiency for the two substrates imply that the mutation at D_211_ affects different steps in the forward and reverse reactions and can have a strong influence on the internal equilibrium between these two competing reactions.

## Discussion

In this study we have examined the determinants of pyrophosphorolysis of a retrotransposon reverse transcriptase. By carrying out a series of reactions using different, but related, partially double stranded substrates we have observed several factors that influence the ability of the enzyme to remove terminal primer bases in the presence of PPi. Our initial substrate (a 14 base recessed 3′ end plus a 14 base double stranded region) was only poorly excised and only a single base was generally removed. Either reduction of the recessed end or an increase in the double stranded primer/template region increased pyrophosphorolysis and made it more processive. In particular, the same 14 base recessed end, with a double stranded region extended to 24 bases, had a much greater capacity for pyrophosphorolysis than the original substrate (Fig. 4 and Fig. S2). In our earlier study [12] we examined dissociation of the enzyme from the 14/28 primer/template, and found a much higher affinity for Ty1 RT (0.06 min^−1^) than for a retroviral RT that we measured concurrently (5.5 min^−1^). This suggests that the limited pyrophosphorolysis observed for the smaller substrate is not due to poor binding. Instead it is more likely that the 14/28 substrate assumes a conformation at the polymerase active site incompatible with pyrophosphorolysis. Even a single additional primer strand base greatly improved pyrophosphorolysis. Studies of the elongation complex of *T. thermophilus* RNA polymerase have shown that a single base pair addition or subtraction to the primer/template complex has marked consequences on the complex conformation and its ability to support pyrophosphorolysis [23].

**Figure 7:**
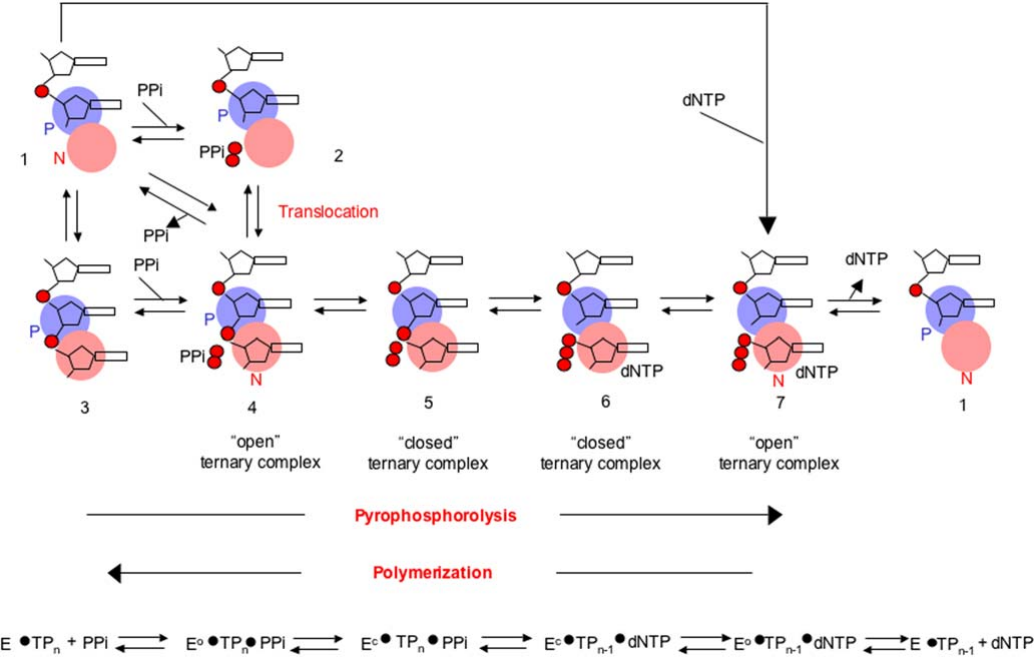
Scheme for phosphoryl transfer reaction by polymerases, from the perspective of pyrophosphorolysis. The polymerase active site contains both a P site (blue circle) in which the 3′ primer-end resides during polymerization, and an N site (pink circle) in which the templated dNTP resides during polymerization. Translocation is required for processive polymerization or pyrophosphorolysis and refers to movement of the 3′ primer-end between the P and N sites (cartoons 1–4). For pyrophosphorolysis to occur, both the 3′ primer-end and a PPi molecule must be correctly positioned in the N site (cartoon 4). It is not known how binding of PPi affects the equilibrium of the 3′ primer-end between the P and N sites (cartoon 2 vs. 4), or whether PPi-binding affinities are affected by the location of the 3′ primer-end (cartoons 1 to 2 versus 3 to 4). Once a ternary complex forms between enzyme, primer template and PPi (cartoon 4), a conformational change is postulated (cartoon 5) that favors the chemical step of nucleophilic attack by the PPi of the 5′ phosphodiester bond in the terminal templated nucleoside monophosphate, and the breakage of the phosphodiester bond to form a dNTP and a new 3′ OH in the P site (cartoon 6 ). Subsequent conformational change in the enzyme (cartoon 7) allows removal of the dNTP and subsequent translocation of the new 3′ primer-end from the P site to the N site (cartoons 1–4).

As shown in Fig. 7 (and discussed below), while the primer 3′ end at the P site can lead to polymerization, the primer-end must be at the N site for pyrophosphorolysis to occur, and this may be disfavored with short substrates. Interestingly, even with processive pyrophosphorolysis of longer primers, the reaction appeared to stop beyond a primer length of 14, further indicating an impediment to continued reaction past this substrate. The RNAse H active site for Ty1 RT has been mapped to between 14 and 15 bases from the 3′ end of the primer terminus in the polymerase active site (as opposed to 18 bases in retroviruses) [24], [25]. Given our previous genetic results on interactions between the Ty1 RT polymerase and RNAse H active sites [10], it is plausible that suboptimal positioning of the substrate at the RNAse H active site could influence the conformation at the polymerase active site in ways that disfavor pyrophosphorolysis. For example, we found that while blunt ends were very good substrates for processive pyrophosphorolysis (Fig. 3 and Figs. S1 and S2), a terminal mismatch or a 3′ extension eliminated the activity. In each case, the primer-end needs to be base paired to the template strand to be in a proper conformation. With a blunt end, there is no extended single stranded template sequence to help position the primer terminus at the P site, and this could facilitate translocation to the N site and subsequent pyrophosphorolysis (Fig. 7).

We speculate that pyrophosphorolysis takes place by at least a 3 step mechanism (Fig. 7). First, both PPi and the 3′ primer-end form a complex at the N site (structures 1–4). Second, a conformational change takes place that brings the primer-end in close proximity with PPi (structure 5). This change from an “open” to a “closed” conformation is analogous to the pre-chemical isomerization steps during polymerization observed for HIV-1 RT, DNA polymerase β and other DNA and RNA polymerases [19], [26]-[28]. Third, the phosphoryl transfer reaction occurs to generate a dNTP at the N site (structure 6). This would need to be followed by release of the dNTP (structures 7 and 1) for any additional PPi to enter the N site.

By carrying out pre-steady state kinetic analysis of the two enzymes, we have identified a source of difference between the WT and mutant enzyme that could contribute to their *in vitro* and *in vivo* behaviors. The WT Ty1 RT has an apparent *K_d_* for PPi of ∼7 mM, similar to the calculated *K_d_* s of several other polymerases examined by related methods [29]–[33]. This millimolar range *K_d_* means that under physiological conditions it is unlikely that PPi is bound at the active site, relative to dNTPs, which have *K_d_*s in the micromolar range. Therefore it was unexpected that the D_211_N mutant had an apparent *K_d_* for PPi in the micromolar range, lower than any other reported polymerase. Since we are determining *K_d_* for PPi indirectly, by measuring product formation as a function of PPi concentration, one reason for the lower PPi *K*_d_ of D211N mutant is its slower chemical step relative to that of the WT enzyme. The *k_pyro_* of the mutant enzyme is ∼320 fold slower than that of the WT. Due to the slower chemical step, PPi bound enzyme intermediates accumulate in the mutant enzyme reaction, but not in the WT. Thus, the apparent *K_d_* in the case of the mutant would correspond to the equilibrium among structures 1 through 4, which reflects the true binding affinity. In other words, the PPi *K*_d_ of the mutant is more close to the “true” *K*_d_ and the PPi *K*_d_ of the WT is close to its *K*_m_.

The difference in the apparent *K_d_* for PPi for WT versus D_211_N mutant RTs, despite their similar *K_d_*s for dNTP, could relate to the potential distortions of the active site architecture in the mutant enzyme due to the D_211_ to N_211_ substitution, its effect on metal binding, and its effect on PPi binding. Our previous work, as well as crystallographic studies of HIV-1 RT, suggests that the D_211_ side chain is more likely involved in the chemical steps and the coordination of the “A site” metal ion, than in the “B site” metal ion which is involved in binding the triphosphates of the dNTP, and presumably, the PPi after chemistry. Modeling studies of the DNA polymerase β active site based on recent high resolution structures suggest that during formation of the transition state, the proton from the primer 3′ hydroxyl is transferred to form a distorted H bond between two aspartates, equivalent to our D_211_ and D_210_, and that this interaction could drive subsequent release of both PPi and the catalytic Mg^2+^ [34], [35]. This implies that a relay of interactions links D_211_ and PPi and that alteration of that relay by N_211_ in the mutant enzyme could affect the apparent affinity of PPi for the enzyme.

The fate of PPi at the active site has become a major focus of attention in models for translocation of polymerases along a template. These models have been made for RNA polymerases in particular, but the basic concepts should apply to all polymerases. One model, referred to as the “power stroke” mechanism, posits that the energy of cleavage of the dNTP results in a change in active site conformation that favors translocation [36], [37]. Yin and Steitz propose for T7 RNA polymerase, that the release of PPi produces the conformational change resulting in translocation [38]. Sarafianos *et al*. propose that movement of the negatively charged loop formed by the HIV1 RT equivalents of D_210_ and D_211_, observed pre- and post- translocation, provides a “springboard” for translocation [19]. A second model, termed “Brownian-ratchet motion” proposes that the primer-end moves back and forth between P and N sites, and that addition of dNTP serve to ratchet the primer-end in the P site where polymerization can occur [39], [40]. In either case, the apparent tighter binding of PPi to the active site of the D_211_N mutant could push the equilibrium more toward the reverse reaction, and impede processive synthesis *in vivo*. Further, since PPi and dNTP reside at the same binding site, PPi might more effectively compete with dNTP in the mutant enzyme. This could explain our previous observations about the *in vitro* polymerization processivity for the two enzymes. On short substrates the two enzymes behaved similarly, but on longer substrates the WT enzyme was much more processive in the presence of Mg^2+^ [12]. Accumulation of PPi during processive synthesis could have inhibited further polymerization in the mutant enzyme. Future analysis of polymerization and processivity under competitive conditions will help to better understand this relationship.

## Materials and Methods

### Plasmids and Strains

The plasmid p6H Ty1 IN-RT-RH (AGE 2186) contained WT Ty1 RT–RH plus a 115 amino acid contiguous C- terminal portion of Ty1 integrase fused to the N-terminus of the RT-RH domain, all preceded by 6-histidine tag and was kindly provided by Dr. F. X. Wilhelm (IBMC, Strasbourg). Construct of the analogous mutant expression plasmid strain AGE 2352, with a D_211_N polymerase active site mutation has been described [12]. The WT and mutant plasmids were transformed into *E. coli* expression strain M15 containing pREP4 (from Qiagen) to generate AGE2193 and AGE2354 respectively [12]. The two enzymes are expressed at similar levels in *E. coli*.

*Reagents-*Denaturing acrylamide gel solutions were from National Diagnostics. Molecular size markers for DNA were from New England Biolabs. The ultra pure dNTPs were from Amersham Pharmacia Biosciences, Inc. Radiolabeled nucleotides were from Perkin Elmer Life Sciences. PAGE purified DNA oligonucleotides were from Integrated DNA Technologies, Inc. Nucleotide removal kit was from Qiagen and P-30 columns were from BioRad. Sodium pyrophosphate, magnesium chloride and manganese chloride were from Fisher Scientific and other chemicals Tris-HCl, NaCl, dithiothreitol and glycerol etc. were from Sigma chemical Co.

### Expression and Purification of Recombinant Ty1 RT

WT and mutant Ty1 RTs containing hexahistidine tags were expressed in E. coli strain M15 [pREP4] (Qiagen) purified by Ni^2+^-nitroloacetic acid-agarose (Qiagen) affinity chromatography as described [12].

### 5′-^32^P Labeling of oligonucleotides

DNA oligonucleotides were 5′ end-labeled using [γ-^32^P] ATP and T4 polynucleotide kinase. Unincorporated nucleotide was removed with nucleotide removal kits (Qiagen) or by P-30 spun columns (Biorad).

### Denaturing gels

Reaction samples were stopped by mixing with loading buffer (95% formamide, 35 mM EDTA [pH 8], 0.1% bromophenol blue, 0.1% xylene cyanol). Samples were heated to 95°C for 5 min prior to loading 7 microliters on a 1x TBE (0.089 M Tris-HCl, 0.089 M boric acid and 0.002 M sodium EDTA buffered at pH 8.5), 7 M urea gel containing the appropriate percentage of polyacrylamide. Electrophoresis was performed in 1×TBE buffer at ∼1–1.2 watts/cm. Gels were visualized with a Storm 860 PhosphorImager and quantitated using the Image Quant 1.2 software (Molecular Dynamics).

### Excision Assay with DNA Templates

In a typical assay, a DNA/DNA template/primer was prepared by annealing a 28-mer plus-strand sequence from the polypurine tract region of Ty1 RT (5′-ATT ACA TTA TGG GTG GTA TGT TGG AAT A -3′, where the polypurine tract is underlined) or an equivalent 28′-mer plus-stand sequence with a ‘g’ at the 3′ end, (to generate a mispaired end) with the otherwise complementary 14 to 28-mer primer whose 5′-end is ^32^P-labeled (Fig. 1, RAG 998 and RAG 1109). Additional substrates used throughout this study are described in Fig. 1.

DNA substrate and enzyme were prepared in the reaction buffer (17 mM Tris-HCl (pH 7.5), 17 mM NaCl, 1 mM dithiothreitol and 20% glycerol) and then pre-mixed with either 10 mM MgCl_2 _or 2 mM MnCl_2_ and a PPi donor (sodium pyrophosphate or NTPs) in the same buffer. The separate mixes were pre-warmed at 22°C for 10 minutes and were then combined in equal volumes to start the reactions at 22°C. Unless specified in the results and figure legends section, an assay mixture contained 40 nM template-primer, ∼20 nM of active WT or mutant D_211_N Ty1 RT, with PPi donor and MgCl_2_ or MnCl_2_ at specified concentrations. Reactions were incubated at 22°C for specified times, and then terminated by the addition of loading buffer, denatured and separated by electrophoresis in 7 M urea-17% polyacrylamide gels. The amounts of the products (i.e. bands smaller than the end-labeled primer) were determined relative to total labeled products seen using a phosphorimager, after subtracting the background from the – PPi lane.

### Pyrophosphorolysis Processivity Assay

The pyrophosphorolysis reactions were carried out by pre-incubating the end labeled 24-mer/28′-mer substrate (RAG*1249/RAG 1109, Fig. 1), (40 nM) with WT or D_211_N Ty1 RT in the reaction buffer for 60 min at 22°C. Reactions were initiated by adding a mixture containing 1 mM sodium pyrophosphate with 10 mM Mg^2+^ or 2 mM Mn^2+^ along with or without an unlabeled 8 µM of 24-mer/28′-mer DNA/DNA substrate trap (at a final concentration of 200 fold more than the labeled primer/template). Reactions were terminated after 1 hour by adding loading buffer. A trap effectiveness control was also carried out where the reaction was initiated by adding PPi with Mg^2+^ or Mn^2+ ^to a mix containing enzyme, trap and primer/template (where enzyme was added to the labeled substrate in the presence of trap and then pre-warmed along with the two). The terminated processivity reaction and control reactions were resolved by 17% polyacrylamide-urea denaturing gel electrophoresis and analyzed as above.

### *K_d_* and *k_pyro_* determination -

The pre-steady-state kinetic parameters for pyrophosphorolysis in the presence of sodium pyrophosphate and 20 mM Mg^2+^ by the WT and the mutant enzymes were measured as reported by Patel et al. [41] with some modifications. Reactions were carried in enzyme excess by rapid quench analysis [42] for WT using a KinTek RQF-3 Rapid Quench Flow apparatus, and manually for the D_211_N mutant. Protein concentrations for both enzymes were determined at OD _280 _in a 1 cm cell in the presence of 8 M urea, (extinction coefficient for the 76. 861 kDa protein = 86,390/cm M). Enzymes were used at final concentrations of 547.92 nM (WT) and 365.34 nM (mutant), assuming that the enzyme is a monomer (F.X. Wilhelm, unpublished results) which were equivalent to active concentrations of 40.46 nM for WT and 40.10 nM for D_211_N as determined by 3 minute extension reactions at 200 micromolar dATP [12]. DNA/DNA template/primer substrate was prepared by annealing a 28′-mer plus-stand sequence from the polypurine tract region of Ty1 RT (RAG 1109) with a complementary 15-mer (RAG 1316) at a template/primer ratio is 0.85/1, to generate the following substrate with a mispair at the 3′ end of the template to avoid forward reaction due to the other end pyrophosphorolysis:
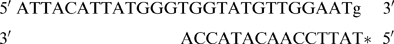
Two separate mixes were made. One was a 2X mix of DNA substrate, PPi and enzyme prepared in the reaction buffer (17 mM Tris-HCl (pH 7.5), 17 mM NaCl, 1 mM dithiothreitol and 20% glycerol) along with different sodium pyrophosphate concentrations and was loaded in one syringe. The second mix consisted of 2X divalent cation (MgCl_2_) in the same buffer and was loaded in the second syringe. These separate mixes were pre-warmed at 22°C for 10 minutes and were then combined in equal volumes to start the reactions at 22°C. The assay mixture contained 20 nM template-primer, ∼40 nM of active WT or mutant D_211_N Ty1 RT, 20 mM MgCl_2_ and various concentrations of sodium pyrophosphate. Samples were taken at several time points and quenched with EDTA, at a final concentration of 35 mM. For D_211_N mutant Ty1 RT, reactions were carried out manually under the same conditions. Products were denatured and resolved in 17% denaturing gels, scanned and then analyzed. Amounts of the excised products (<15-mer) were determined relative to total labeled products. The time course of a single turnover for individual [PPi] was fitted to an exponential equation (Equation 1). The calculated rates were plotted against respective [PPi] to fit this data to a hyperbolic equation (Equation 2).

*Equation 1:*

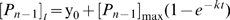
where k is the observed rate per second, [P_n-1_]_t_ is the amount of product at a given time, t, [P_n-1_]_max_ is the maximum amount of product formed is in nM and y_o_ is the y intercept.

*Equation 2:*

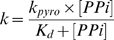



## Supporting Information

Figure S1 Pyrophosphorolysis by WT and D211N Ty1 RT of a recessed primer terminus in the presence of a mismatched other end eliminates forward reaction. Time course of pyrophosphorolysis for WT or mutant D211N Ty1 RT were carried out under same conditions using 5′ 32P-end labeled 14-mer/28′-mer substrate (RAG*998/1109, Fig. 1) in the presence of 10 mM Mg2+. A, shows time course of WT reactions in seconds in the presence or absence of PPi. B, represents time course of D211N reactions with or without PPi. Control reactions are in the absence of Mg2+ or enzyme.
(0.09 MB TIF)

Figure S2 Effects of different primer-ends and double strand length on pyrophosphorolysis. Pyrophosphorolysis reactions by WT were carried out for 60 minutes in the presence of either 10 mM Mg2+ or 2 mM Mn2+. A, shows relative positions of substrates 1–7 graphically. Substrates 1–7 are 5′ 32P-end labeled (1; RAG*1253/1050, 2; RAG*1249/1109, 3; RAG*998/1109, 4; RAG 998/*928, 5; RAG 955/*928, 6; RAG 831/*928, 7; RAG 952/*928, Fig. 1). B, shows WT reactions with different substrates in the presence of Mg2+ with or without sodium pyrophosphate. C, represents reactions with Mn2+. D, shows bar graph of I cleaved products/I total bands, black bars are for the reactions with Mg2+ and grey bars are for the reactions with Mn2+.
(0.25 MB TIF)
