# Supplementary figures and images for: Kinetic Pathway of Pyrophosphorolysis by a Retrotransposon Reverse Transcriptase
Source: PLoS One. 2008 Jan 2;3(1):e1389. doi: 10.1371/journal.pone.0001389 (PMC2148107; doi:10.1371/journal.pone.0001389)

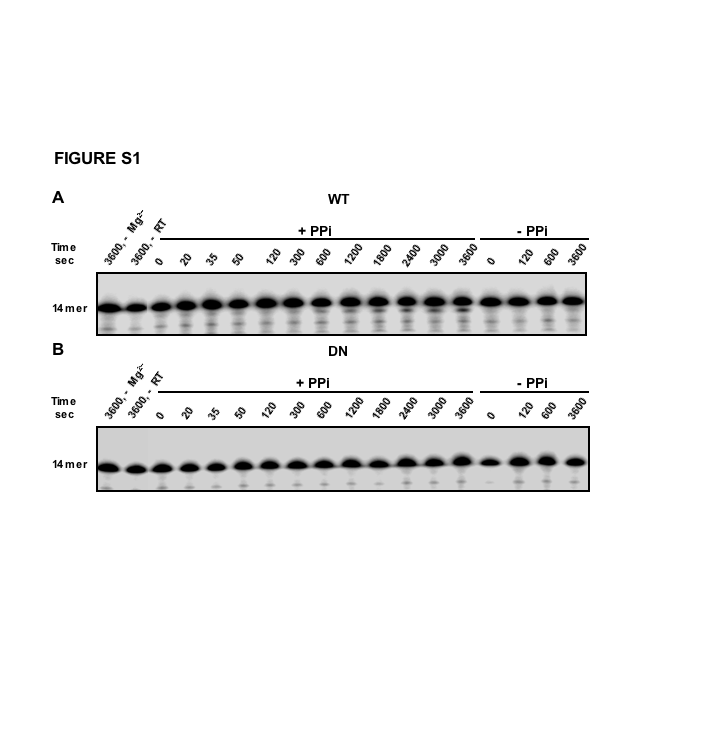

Supplement: Figure S1 — Pyrophosphorolysis by WT and D211N Ty1 RT of a recessed primer terminus in the presence of a mismatched other end eliminates forward reaction. Time course of pyrophosphorolysis for WT or mutant D211N Ty1 RT were carried out under same conditions using 5′ 32P-end labeled 14-mer/28′-mer substrate (RAG*998/1109, Fig. 1) in the presence of 10 mM Mg2+. A, shows time course of WT reactions in seconds in the presence or absence of PPi. B, represents time course of D211N reactions with or without PPi. Control reactions are in the absence of Mg2+ or enzyme. (0.09 MB TIF) [file pone.0001389.s001.tif]

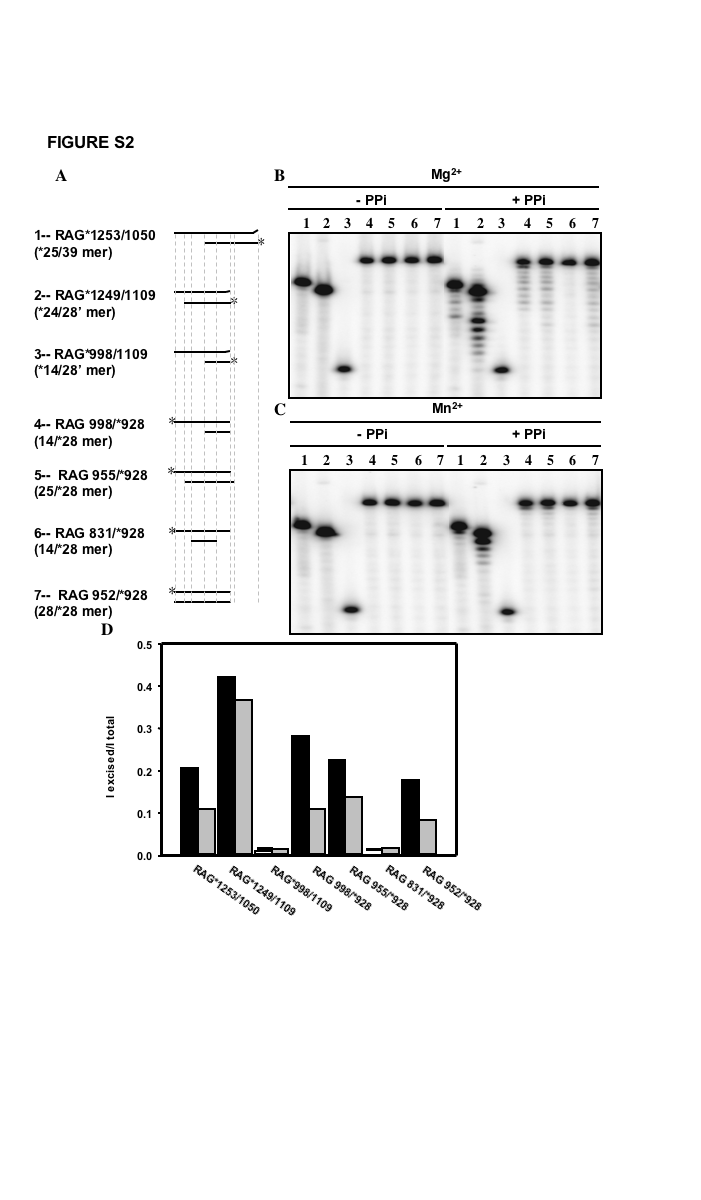

Supplement: Figure S2 — Effects of different primer-ends and double strand length on pyrophosphorolysis. Pyrophosphorolysis reactions by WT were carried out for 60 minutes in the presence of either 10 mM Mg2+ or 2 mM Mn2+. A, shows relative positions of substrates 1–7 graphically. Substrates 1–7 are 5′ 32P-end labeled (1; RAG*1253/1050, 2; RAG*1249/1109, 3; RAG*998/1109, 4; RAG 998/*928, 5; RAG 955/*928, 6; RAG 831/*928, 7; RAG 952/*928, Fig. 1). B, shows WT reactions with different substrates in the presence of Mg2+ with or without sodium pyrophosphate. C, represents reactions with Mn2+. D, shows bar graph of I cleaved products/I total bands, black bars are for the reactions with Mg2+ and grey bars are for the reactions with Mn2+. (0.25 MB TIF) [file pone.0001389.s002.tif]
